# Redevelopment of urban brownfield sites in China: Motivation, history, policies and improved management

**DOI:** 10.1016/j.eehl.2022.04.005

**Published:** 2022-05-21

**Authors:** Yiming Sun, Hong Li, Shuo Lei, Kirk T. Semple, Frederic Coulon, Qing Hu, Jingyang Gao, Guanlin Guo, Qingbao Gu, Kevin C. Jones

**Affiliations:** a State Key Laboratory of Environmental Criteria and Risk Assessment, Chinese Research Academy of Environmental Sciences, 100012, Beijing, PR China; b Lancaster Environment Centre (LEC), Lancaster University, Lancaster, LA1 4YQ, UK; c Centre for Ecology and Hydrology, Library Avenue, Bailrigg, Lancaster LA1 4AP, UK; d State Environmental Protection Key Laboratory of Regional Eco-process and Function Assessment, Chinese Research Academy of Environmental Sciences, 100012, Beijing, PR China; e School of Water, Energy and Environment, Cranfield University, MK43 0AL, UK; f Southern University of Science and Technology, 518055, Shenzhen, Guangdong, PR China; g Beijing Huanding Environmental Big Data Institute, 100083, Beijing, PR China; h Technical Centre for Soil, Agricultural and Rural Ecology and Environment, Ministry of Ecology and Environment, 100012, Beijing, PR China

**Keywords:** Brownfield, Urban soils, Management, China, USA, UK

## Abstract

Rapid urbanisation in China has resulted in an increased demand for land in towns and cities. To upgrade and modernise, China has also moved many major industries from urban centres to less populated areas. With the high economic value of urban land, the transformation and utilisation of brownfield areas have become important economically and socially. The Chinese government has recognised the need for strong frameworks to safeguard soil and groundwater quality, with brownfield sites a key category for management. Strong scientific, regulatory and decision-making frameworks are needed and being adopted to ensure practical, careful and wise use of central and localised government resources, to manage the reuse and regeneration of these brownfield sites. This paper reviews the context, policies and management procedures of developing brownfield sites in countries with a history of brownfield management and discusses China’s current situation and priorities for brownfield governance and redevelopment. These include (1) clarification of brownfield site soil contamination risk control standards and risk assessment procedures, (2) the responsibilities of different national and local agencies, (3) the establishment of a national expert committee to advise on best practices, policy and process, (4) the use of registered brownfield databases at national, provincial, municipal and county levels, and (5) the set up of soil pollution prevention fund at the provincial level.

## Highlights

• China is setting strong frameworks to safeguard soil and groundwater quality.
• Brownfield soil quality standards and risk assessment procedures are needed.
• Responsibilities of national and local level agencies are being prioritised.
• Brownfield databases at national, provincial, municipal and county levels need setting.

## Graphical abstract

**Figure:**
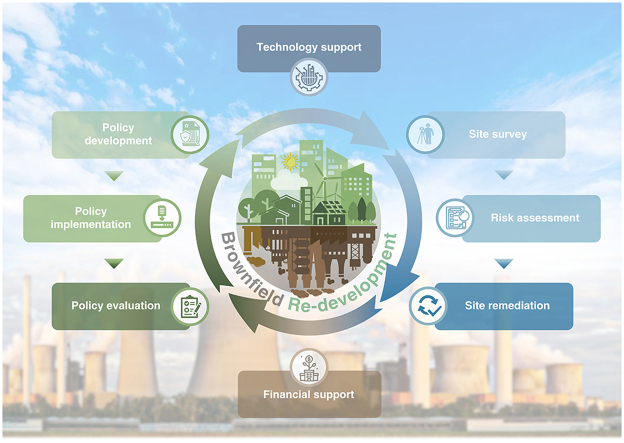

## 1 Introduction

### 1.1 Urbanisation in China

Over the past 40 years of reform and development, China has undergone remarkable economic growth. The scale of China’s urbanisation and the number of growing large metropolitan regions where this urbanisation is concentrated is globally unprecedented [1]. Many industrial facilities in cities have been relocated or closed, leaving behind derelict, underused and abandoned land contaminated by former industrial activities. The continuous outward shift of urban boundaries, primarily through the expropriation of surrounding rural land and its integration into urban areas, results in new urban and peri-urban expansions increasing the fragmentation of the landscape [2]. During the first industrialisation period, the decline of traditional industries and the relocation of a large number of factories led to a large number of unused and abandoned sites in the city [3]. The consequence of post-urbanisation also occurred in western countries, such as the United Kingdom and the United States. As the first country to industrialise, the United Kingdom began to pay attention to this problem as early as the 1970s. Similarly, after a series of solid waste pollution incidents, the United States also paid attention to this matter. How to reuse such sites was a significant challenge for these countries at the time.

With the rapid development of urbanisation, land resources are also becoming increasingly valuable. Despite the differences in urban structures, both China and other countries face an ongoing trend toward urbanisation and increasing stock of marginal land [4,5]. A shared endeavour is needed to promote the development and utilisation of the vacated and abandoned land in China, often called ‘brownfield sites’ in western countries. This has been identified as a priority for environmental regulation and management in China [6,7,8]. China is engaged in serious efforts to implement brownfield redevelopment on a large scale. The Chinese government intends to initially introduce the brownfield redevelopment framework on a smaller scale through a number of pilot studies to establish a better basis for assessing its large scale and full coverage in the longer term. This study highlights the needs and opportunities arising from rapid urbanisation and the changes in land use resulting from industrial transformation, which has left a legacy of polluted industrial, commercial and marginal land areas in China. This study also highlights the remaining challenges and opportunities for the brownfield market in China.

### 1.2 The brownfield concept

The term ‘brownfield’ is believed to have been used first in the Comprehensive Environmental Response, Compensation and Liability Act (CERCLA) in the United States in 1980 [9]. The Superfund Program was designed in the Act to solve the problem of the locally and nationally significant public health and environmental dangers caused by heavily contaminated properties [10]. In 1993, The Brownfields Initiative was launched to redevelop abandoned, unused or underused industrial and commercial sites where expansion or redevelopment was complicated by real or perceived environmental contamination. In 1995, the US Environmental Protection Agency (USEPA) provided small amounts of seed money to local governments to launch hundreds of two-year brownfield pilot projects and develop guidance and tools to help states, communities and other stakeholders clean up and redevelop brownfields sites. In 2002, the Small Business Liability Relief and Brownfield Revitalisation Act was released to boost funding for the assessment and clean-up of brownfields, enhance roles for state and tribal response programs, and clarify Superfund liability. While these Brownfield and Superfund Programs were both about contaminated site management, they differed in the extent of site contamination. Superfund sites were national priority sites with the most serious pollution, while brownfield sites were abandoned or unused sites, generally in urban areas, where reuse was planned. Around 1990, the term ‘brownfield’ also appeared in British planning regulations, referring to ‘previously developed land’ as unused or exploitable land, including vacant, abandoned land and currently used land with the potential for redevelopment [11,12,13]. In the United Kingdom, ‘brownfield’ is widely understood to be abandoned or vacant land that can be redeveloped in accordance with planning policies or urban revitalisation goals [14]. In the United States, ‘brownfield’ is generally interpreted as occupied or contaminated land [15,8,16]. Alker et al. [17] proposed a comprehensive definition of brownfield—any land or premises that has previously been developed and is not currently fully in use, although it may be partially occupied or utilised. It may also be vacant, derelict or contaminated. Therefore, a brownfield site is not available for immediate use without intervention [17]. Other useful terms include derelict land, i.e., land damaged by industrial or other development that is incapable of beneficial use without treatment [18] and contaminated land—an indication of the presence of some biological, chemical or physical hazard on or within a site that would require some treatment before the site could be reused [17].

**Fig. 1:**
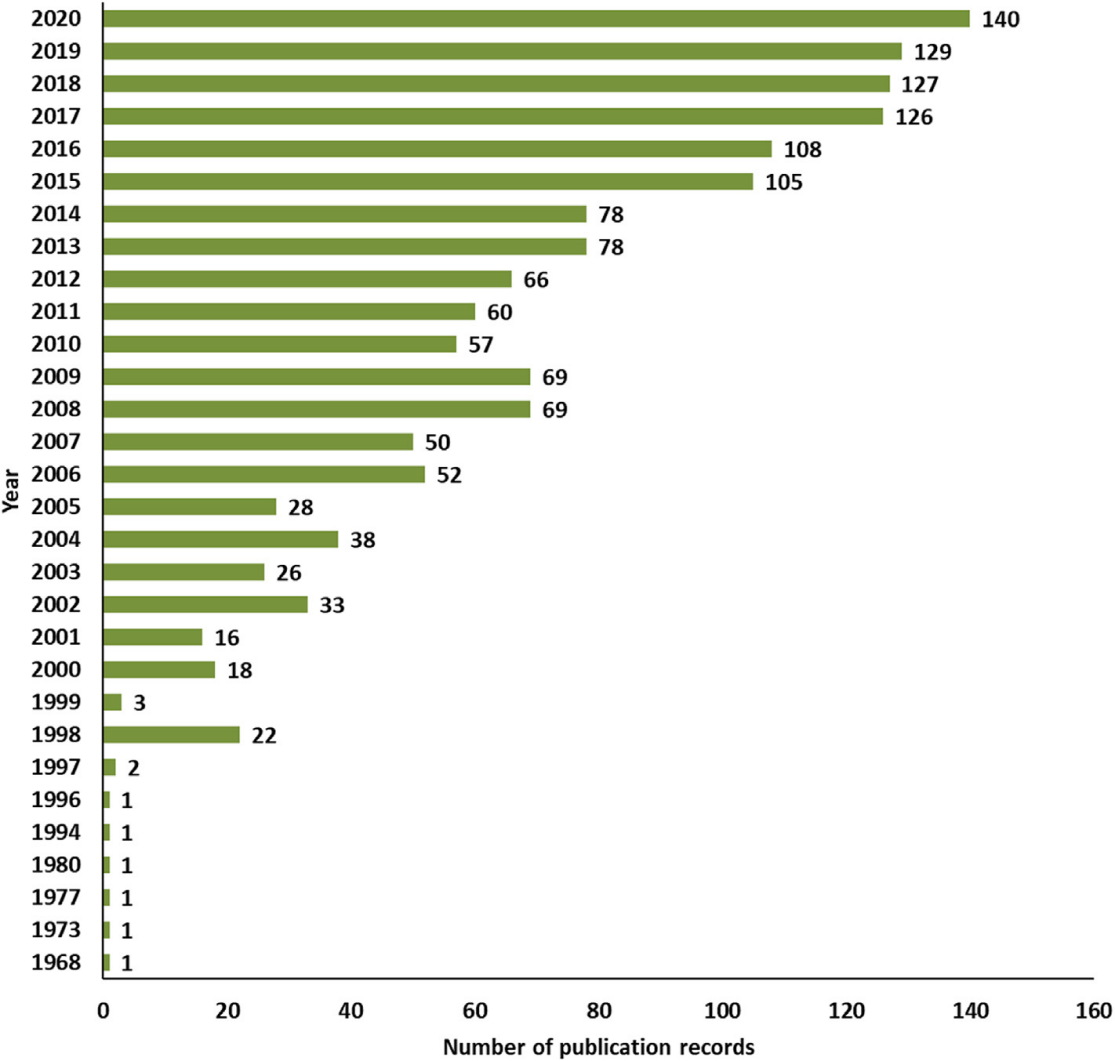
The publication records related to brownfields from 1968 to 2020 in the Web of Science. The searched keyword is ‘brownfield’ in the Web of Science Core Collection.

**Fig. 2:**
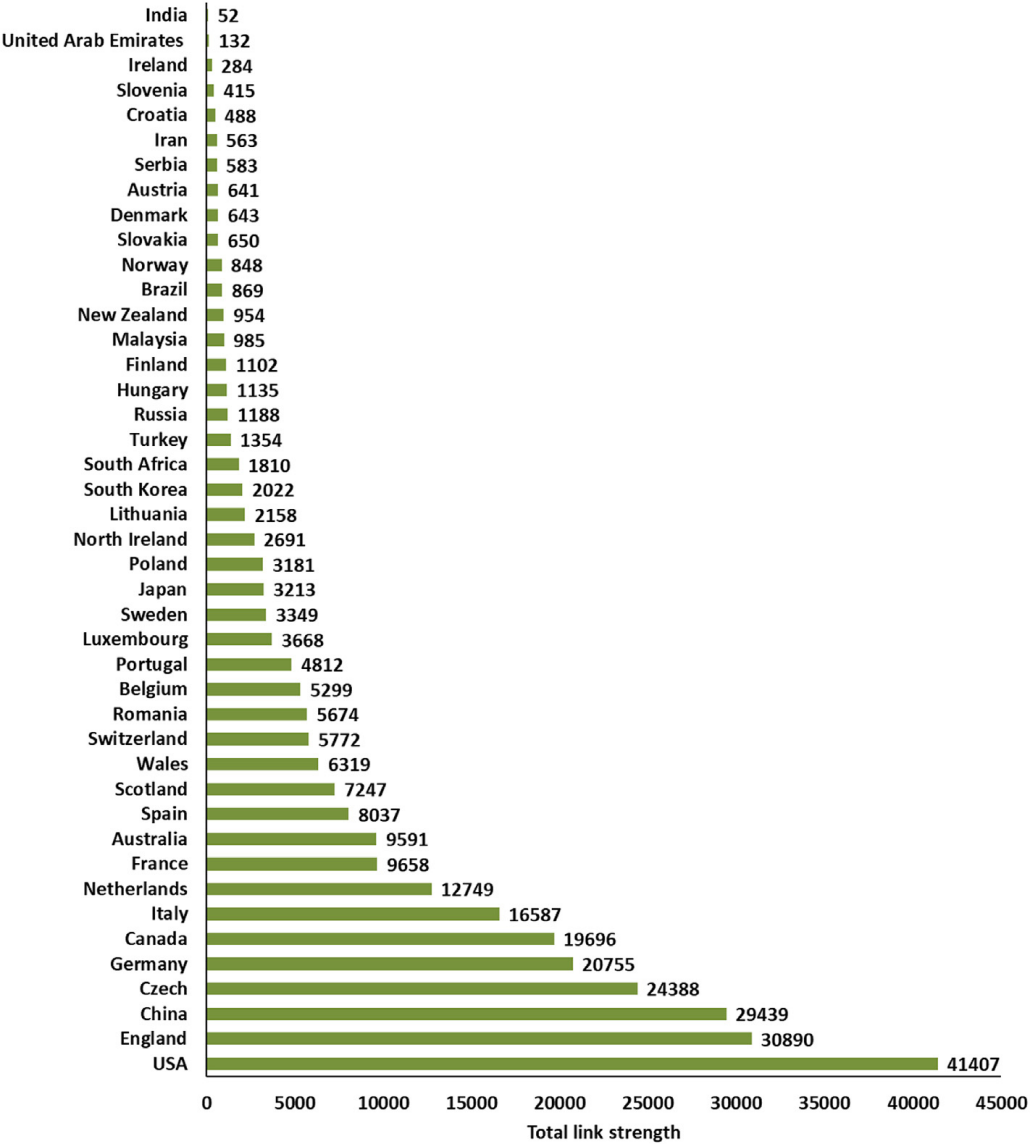
A bar chart of the research relevant to brownfields in different countries from Web of Science. Total link strength refers to the total strength of the country links of a given country with other countries and was generated by VOSviewer software [23].

**Fig. 3:**
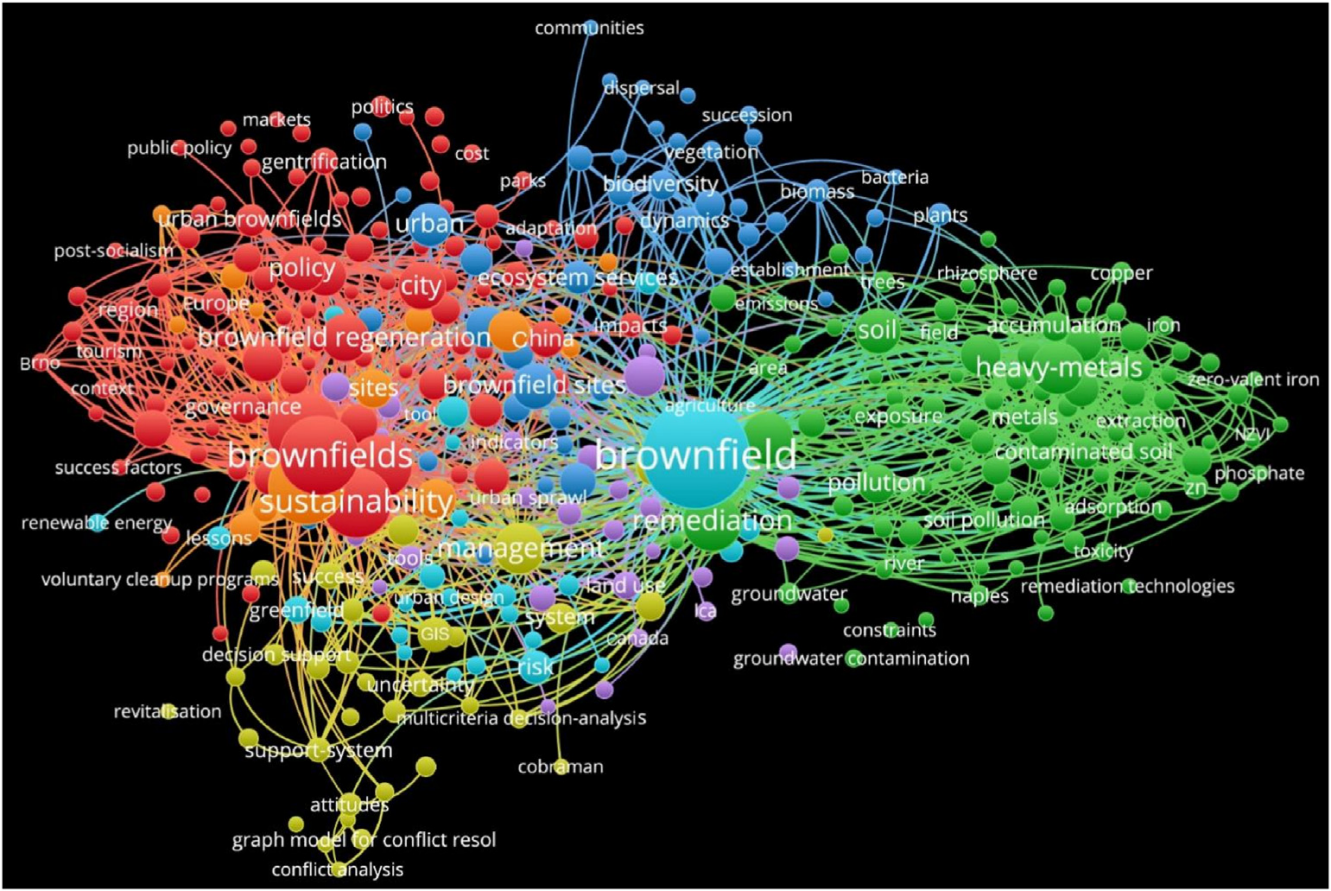
The co-occurrence network map of the research keywords relevant to brownfields from Web of Science, with 1506 publication records downloaded from Web of Science from 1968 to 2020, integrated into this analysis. The colour of a keyword was determined by the cluster to which the keyword belonged. Lines between keywords represent links, and 1000 lines were displayed at most to avoid the overlapping of keywords, representing the 1000 most occurrences between keywords. The size of the label and the circle of a keyword were determined by the occurrence of the keyword. The higher the occurrence of a keyword, the larger the label and the circle of the keyword.

In order to clearly understand the overall status of brownfield research around the world, a bibliometric analysis of literature was performed here. By setting the search subject term as ‘brownfield’ in the Web of Science Core Collection and the document type as ‘Article’, with a search time span from 1968 to 2020, a total of 1506 papers were obtained for analysis. Based on the trends of publications, two stages were clearly shown (see Fig. 1). In the initial phase, 51 articles relating to brownfields were published between 1968 and 2000. This was followed by a huge expansion between 2001 and 2020, with 1455 publications over 20 years. The 1506 publications relating to brownfields were from 68 countries, with mostly the United States, the United Kingdom, China, the Czech Republic, Italy, Germany, Canada, etc (Fig. 2). Three main groups of research keywords are highlighted in Fig. 3, namely ‘Brownfield redevelopment or regeneration’, ‘Brownfield remediation’ and ‘Heavy metal contamination or management’.

### 1.3 Brownfields in China

In China, the term ‘brownfield’ was first mentioned by Ref. [19] when introducing the US brownfield definition and regulations and how they can be applied to real case studies in China. According to the World Bank’s ‘Waste Management in China: Problems and Suggestions’ issued in 2010, there were ‘at least 5000 brownfield sites’ in China. In reality, this estimate was likely to be 1–2 orders of greater magnitude [20].

The national soil survey published in April 2014 by the Ministry of Environmental Protection of China (MEP, currently the Ministry of Ecology and Environment of the People’s Republic of China) and the Ministry of Land and Resources of China (currently the Ministry of Natural Resources of the People’s Republic of China) revealed the significant challenges that China was faced with in soil pollution. Extrapolation of the soil survey indicated that substantial areas (36% of sampling points) within the vicinity of industrially contaminated sites were potentially contaminated [21].

In 2008 the MEP issued the ‘Opinions on Strengthening the Prevention and Control of Soil Pollution’ and put forward corresponding action measures, including (1) completing the investigation of the soil pollution situation in a ‘comprehensive’ way, (2) establishing a soil environmental monitoring network, (3) compiling and completing national and local soil pollution prevention and control plans, (4) establishing policies and laws for soil pollution prevention and control, and (5) establishing a management system framework, such as laws and regulations [22].

China’s State Council released the ‘Action Plan on Prevention and Control of Soil Pollution (10-Chapter Soil Pollution Action Plan)’ in 2016. This was heralded as a critical development in identifying and prioritising wise use and management of China’s soil resources. The 10-Chapter Plan presents the requirements, work plan and main goals of China’s national soil contamination prevention priorities [7] (Table S1).

The Chinese authorities also committed over 30 billion RMB within the national 12th Five-Year Plan to address soil pollution, along with a specific plan for its prevention and control, which came into force during the period of the 13th Five-Year Plan (2016–2020). Along with the development of the nation’s first specific national law on the control and prevention of soil pollution being drafted by the MEP, the implementation of this plan demonstrated the commitment to long-term soil management and regeneration of industrialised sites. China has set very ambitious targets for a high percentage of contaminated sites to be used by 2020 and beyond and established a soil contamination risk control standard system [7]. It will further promote on-site remediation, as well as the opening up of the monitoring services market.

## 2 The experiences of brownfield redevelopment in the United States and the United Kingdom

### 2.1 The development of brownfield management in the United States

In the late 1970s, some contaminated land incidents raised government and public attention in the United States, resulting in the release of the CERCLA in 1980. It required the owners, users and polluters of real estate to bear the consequences of land pollution and cover the cost of land governance through the form of law.

In 1986, the Superfund Amendments and Reauthorisation Act (SARA) updated some provisions:

• emphasised the importance of technological innovation in permanent remediation and remediation of hazardous waste sites;
• ensured that environmental laws and standards of the federal and states governments were taken into account when implementing superfund operations;
• proposed a new executive body and dispute settlement mechanism;
• increased the involvement of state governments in each phase of the superfund plan;
• paid more attention to the human health problems caused by hazardous waste sites;
• encouraged more citizens to participate in the decision-making of the site restoration process;
• increased the investment of trust funds.

As mentioned in Section 2.1, the framework for the rehabilitation of contaminated sites in the United States mainly includes the CERCLA passed in 1980. This bill, often referred to as the ‘Superfund Law’, establishes the ‘polluter pays’ principle, stipulating that different parties (legally defined as ‘potentially responsible parties’) are responsible for remediating historically contaminated sites. In addition, the ‘Superfund Law’ authorises the US Environmental Protection Agency to force any potentially responsible party to pay for the remediation of the site. The sharing of site remediation costs and the sharing of responsibilities would be resolved between potentially responsible parties. However, the CERCLA was criticised for many shortcomings, including lengthy legal proceedings, burdens on small businesses and insufficient participation of state governments and local communities, since the main actions are the responsibility of the Federal government. Furthermore, due to uncertainties with regard to responsibilities and liability, several investors and developers were discouraged from becoming involved, leaving sites empty or undeveloped and eventually becoming brownfields. These shortcomings of the law have gradually been corrected through multiple rounds of amendments and reforms to the Superfund program over the years, including the 2002 Small-Scale Corporate Responsibility Mitigation and Brownfield Revitalisation Act and other brownfield-related projects and plans (Fig. S1). The revised Superfund Law is now welcomed by various stakeholders. These amendments and reforms are practical lessons for brownfield management in developing countries like China. In addition, the lessons learned from the US Superfund Act, including the high cost of remediation of contaminated sites, and the knowledge that scientific management, such as controlling the spread of existing pollution, is more effective than site remediation in many cases.

### 2.2 The development of brownfield management in the United Kingdom

In the United Kingdom, the Interdepartmental Commission for Redevelopment of Polluted Sites (ICRCL) was the first to address the problem of contaminated sites. It is responsible for providing advice and guidance on health hazards caused by the reuse of contaminated sites and coordinating recommendations on remediation measures. The Committee issued Guidelines 59/83 in 1987 to guide practitioners in dealing with different types of hazards and pollution. In 1990, the United Kingdom first legislated to regulate contaminated land by enacting the Environmental Protection Law. In 1998, the National Land Use Database (NLUD) was established and began to identify and address the management of brownfield sites. In the database, land use was divided into 51 categories and began to evaluate the suitability of redevelopment of brownfield sites and other sites. In 2000, the Environment Agency asked local governments to confirm the treatment of contaminated land. The Guidelines 59/83 in 1987 defined ‘trigger values’ (thresholds and action values) of land for different planning purposes, which were officially cancelled by the Department of Environment, Food and Rural Affairs (DEFRA) in 2002. Since 2005, the sustainable development strategy has been highly valued in the planning and development of land in the United Kingdom. The United Kingdom government believes that brownfield governance and redevelopment are key to promoting economic growth and maintaining social development while minimising environmental impact [24] (Fig. S2).

In United Kingdom, in brownfield governance, the government has played a leading role, and the brownfield risk management and restoration policy promoted by it has achieved good results. From 1988 to 1993, 19% of brownfield sites in the United Kingdom were converted into greenfield sites. Brownfield treatment has improved the quality of urban environments and reduced the pressure on rural land development. The NLUD database shows that about 28,810 ha (45%) of brownfield land may be suitable for residential use, so the UK’s brownfield management took the reuse as a starting point, using market drivers to realise its economic benefits. In 1998, the government policy was that 60% of new homes to be built in 2008 or the renovation of existing residences needed to be carried out on brownfields. This goal was achieved ahead of schedule in 2002, and by 2008 this indicator reached 80%.

In general, brownfield governance in the United States and the United Kingdom started early, and the government has played a leading role, effectively coordinating the ecological benefits based on sustainable development and the economic and social benefits based on land redevelopment in brownfield governance. The value concept of brownfield governance has developed from the administration-orientated stage to the economic-orientated stage and then to the environmental justice-orientated development model [25]. In this process, responsibility identification, fiscal and tax incentives and public participation models were key issues in brownfield governance [26]. The transformation of the role of public administration, the upgrading of the understanding of the connotation of brownfields and the refinement of governance policies are important reasons for the success of brownfield governance [[3], [25]]. Specifically, combining the experience of the United States and the United Kingdom, successful brownfield governance [27] has the following features, which are important for China and other countries to consider: (1) established legal and regulatory guarantee systems, (2) attention paid to public participation in the whole process of brownfield governance and remediation redevelopment, and the link of contaminated land reuse and remediation to the planning process, (3) an established brownfield register that regularly publishes brownfield information, and mobilises the enthusiasm of all stakeholders, (4) a funds guarantee system, including financial allocation (national government providing special fund for brownfield redevelopment), tax relief (making full use of market mechanisms, reducing the cost of redevelopment of brownfield sites by private enterprises and encouraging private investment to enter the field of brownfield redevelopment), and (5) ‘polluter pays’ system (units and individuals that cause damage to land and environment are required to assume corresponding responsibilities for pollution control).

## 3 The process of contaminated urban soil management in China

### 3.1 Legal system for brownfield governance and redevelopment

Due to the relatively short development time of China’s industrialisation and urbanisation, less attention has been paid to brownfield issues. At present, there are no policies or regulations specifically for brownfield management and redevelopment, but only some relevant ones. Since the mention of soil control in the Environmental Protection Law of 1989, China has issued about 36 national-level documents related to soil pollution control [28], such as laws, regulations and technical guidelines (see Table S2), of which 17 are related to urban brownfield reuse.

**Fig. 4:**
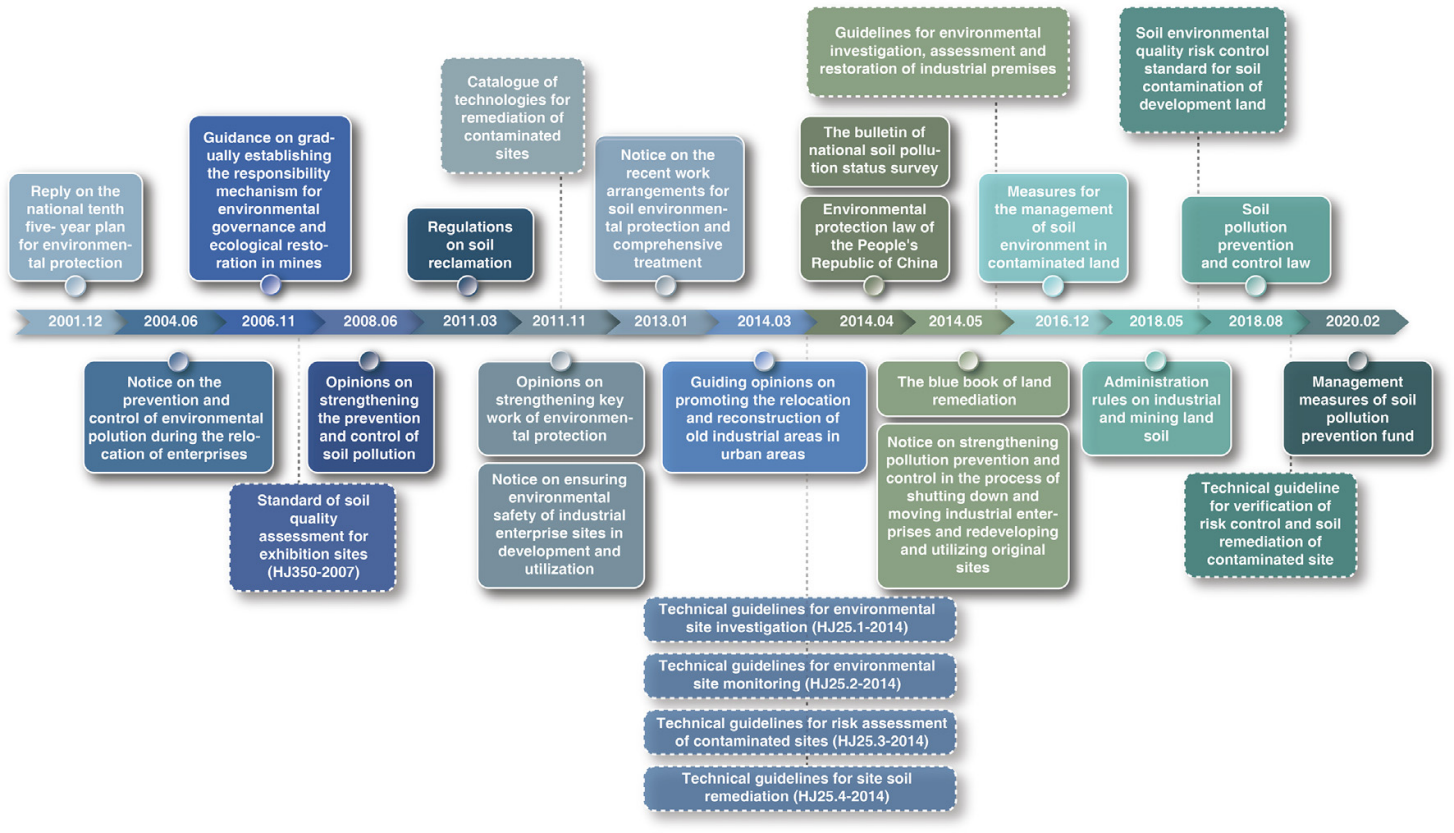
A timeline of contaminated land regulations in China. Box with solid line represents the issued regulations and laws from 2001 to 2020. Box with dash line represents the issued technical guidelines during this period, among which the guidelines coding with HJ were issued by the Ministry of Ecology and Environment of the People’s Republic of China (MEE).

In June 2004, the State Environmental Protection Administration (currently the Ministry of Ecology and Environment of the People’s Republic of China) issued the ‘Notice on Effectively Preventing and Controlling Environmental Pollution in the Process of Enterprise Relocation’, which first raised the issue of soil pollution for soil redevelopment. Fig. 4 details some key steps that followed. By December 2016, the MEP issued the ‘Measures for the Management of the Soil Environment in Contaminated Land’, which stipulated the soil environmental investigation and risk assessment system, the risk management and control system of contaminated land, and the contaminated land governance and restoration system. In the most recent five years, China entered the ‘Policy establishment development period’ of soil contamination management. Many key regulations and laws were issued in 2018–2020, with the establishment of laws and regulations on ‘Industrial and Mining Soil Management Methods’, ‘Soil Pollution Prevention and Control Law’, ‘Land Use Survey Manuals for Key Industries’, ‘Certification Methods for Construction Land Responsible Persons’, ‘Performance Evaluation Methods of the Central Finance Ecological Environmental Protection Special Fund’ and ‘Management methods for soil pollution control funds’. In general, China’s brownfield governance policy development can be divided into three stages: problem outbreak period (2004–2008), policy exploration period (2009–2014) and policy establishment and development period (2015–present). The third stage is coming to an end, and the next steps will see the enactment of the policies trying to solve urban contamination problems. This requires strong policies and laws, together with good knowledge and practical actions at national, regional and local scales.

Land ownership is a major difference when comparing brownfield management in China with that in the United Kingdom and the United States. China’s land ownership is completely state-controlled and individuals or businesses only have the right of land use, with properties typically bought or leased for 40–70 years from the government [29]. That is to say, the government has an absolute control right in brownfield management, financing and supervision, which means state ownership makes the responsibility and management pathways potentially easier for China. Nonetheless, China has many common challenges in brownfield management, including multiple levels of government control and multiple stakeholders, e.g., different Ministries, planning and development offices, expert groups, residents’ groups, etc. China has undergone institutional reforms, optimising and integrating the multiple sectors related to soil, water and marine fields, centralising them into the MEE, and establishing corresponding professional departments and technical support units. For example, in 2018, to prioritise environmental management and coordinate the decision-making processes, the MEE was formed by integrating the environmental management functions of the former Ministry of Land and Resources, Water Conservancy Department, Marine Bureau, Agriculture Department and Development and Reform Commission. Its remit is to exercise the responsibilities of supervising environmental/ecological management in a unified manner, focusing on strengthening the four primary functions of the ecological environment system, namely formulating policies and regulations, monitoring and evaluation, supervision and enforcement and accountability. Other ministries and their responsibilities for environmental protection are as follows:

• The National Development and Reform Commission is responsible for the overall coordination of national-level special planning, regional planning, spatial planning and national development planning. It proposes policies and measures to improve the ecological protection compensation mechanism and comprehensively coordinates the work related to the promotion of environmental protection industries and cleaner production.
• The Ministry of Water Resources is responsible for (1) organising the preparation and implementation of water resources protection plans, (2) guiding the protection of drinking water sources, (3) guiding the development and utilisation of groundwater and the management and protection of groundwater resources, (4) guiding the management, development and protection of important rivers, lakes and estuaries, and (5) guiding the ecological protection and restoration of rivers and lakes, river and lake ecological flow and river and lake water system connectivity.
• The Ministry of Housing and Urban-Rural Development is responsible for (1) guiding the paid transfer, development and utilisation of urban land use rights, (2) guiding the improvement of the living environment in small towns and villages, (3) organising the implementation of major building energy conservation projects, and (4) promoting urban emission reductions.
• The Ministry of Agriculture and Rural Affairs is responsible for taking the lead in organising the improvement of the rural living environment and guiding the environmental management of agricultural production areas and agricultural cleaner production.

Since the founding of the People’s Republic of China in 1949 and the simultaneous abolition of privatisation, there has been no change in the policy for public ownership of land and other natural resources. Article 74 of the General Principles of Civil Law of China states that ‘the collectively owned land belongs to the village peasant collectively in accordance with the law’. According to the newly revised Land Management Law, China adheres to the socialist public ownership of land, i.e., ownership by the whole people and collective ownership by the working people. According to Chinese law, ownership by the whole people means that the right of ownership in state-owned land is exercised by the State Council on behalf of the state. It authorises relevant ministries and subordinate (provincial and municipal) governments to exercise the property rights of natural resources. The central government plays a leading role in implementing, protecting and supervising the property rights and arrangement of natural landscape resources. However, this leadership role has not yet been assessed in terms of the effectiveness of land use management. The government has allocated part of its budget for the maintenance, planning and management of brownfield sites. By contrast, according to the current land use management system, all levels of government (i.e., central, provincial, municipal and local) need to disclose the types and scale of land use to planners, owners and operators. Therefore, the interpretation of ownership and the implementation of land use policies related to ownership may be a major issue for future governance of brownfield sites.

With legislation, there is a solid basis for the construction of an urban soil environmental management system. The legislation needs to be enforced, be workable, fair and just. The United States and the United Kingdom both have specific laws on soil protection. They provide a legal basis for soil environmental protection, stipulate a management system, clarify the rights and obligations of the main body of governance, and urge local governments and their departments to follow the law, thus managing the prescribed steps, methods or procedures. In terms of legislation, countries tend to establish precise procedures with evaluation according to local conditions, implement regulations on urban contaminated sites, and at the same time achieve the goal of improving the effectiveness of urban contaminated sites by gradually improving scientific and technological standards. Therefore, the central government should act as a monitoring body to release the standards of enforcement and supervise its results, while the local governments and agencies need to enforce their power by following the national policies. For this aspect, China issued a law on the prevention and control of soil pollution in August 2018 and implemented it in January 2019. The law aims to protect and improve the ecological environment, prevent and control soil pollution, protect public health, promote the sustainable use of soil resources, promote the construction of ‘ecological civilisation’, and promote sustainable economic and social development [30].

Under the Chinese framework, local governments need to evaluate local soil background levels, conduct risk management and control in high background value areas, promote technical reviews, public participation and information disclosure, etc., to improve the environmental management of local contaminated land as a part of the local government’s soil environmental management. In China, the policy and regulatory framework for brownfield management need to consider China’s national conditions, such as:

1) significant differences in economic and social development levels between different regions;
2) availability of supporting infrastructures, such as landfills and transport, storage and disposal facilities;
3) the level of competence, knowledge and technical skills;
4) length of pollution history, extent and nature of site pollution;
5) the consequences of exposure risks.

Therefore, it seems more prudent to choose regional and phased approaches, based on the national guidelines, to establish a framework for contaminated site management.

### 3.2 The registration information system for brownfield sites in China

The data currently available in China can only be extracted from ‘Waste Management in China: Issues and Recommendations’ published by the World Bank in May 2005, which describes that ‘there are at least 5000 brownfield sites in the country’. So far, no precise national data has been officially released. Therefore, China’s first step in brownfield management is to establish a brownfield registration system to find out the number and pollution status of brownfields as soon as possible. Experience from Western countries indicates that suspected contaminated sites should be investigated and screened, and a professional database should be established. It should hold detailed records of the location, size and nature of potentially contaminated sites. Such information needs to be held locally to inform city planning and development, while information on large, hazardous or priority sites will be needed nationally. It will also help later land users understand its basic conditions and avoid asymmetric information between developers and owners. If an accident occurs during subsequent use, the relevant data provided by the brownfield database can be retrieved to trace responsibilities, and provide land governance information and governance process data for future brownfield pollution control. Based on the brownfield database, brownfield sites can be managed hierarchically, and classification of site management and development can be implemented. China has now begun to instigate such a scheme. In 2016, the MEP released the Measure for the Management of Soil Environment in Contaminated Land. It provides a procedure for suspected contaminated land from definition to supervision. Suspected contaminated land is considered land engaged in production and operation activities in non-ferrous metal smelting, petroleum processing, chemical, coking, electroplating, tanning and other industries, in hazardous waste storage, utilisation and disposal activities. The ministry recommends establishing a national soil environmental management information system for contaminated sites. It requires local environmental protection authorities at or above the county level to organise the construction and application of such systems within their respective administrative areas. The owner and user of the suspected contaminated land must fill in and submit its information and related activities online through the contaminated land information system. The MEE then implements information sharing with the urban and rural planning departments and land and resources departments through the information system. The list of suspected contaminated sites should be regularly updated. The land use right holder is required to carry out site investigation, risk assessment and remediation evaluation procedures in accordance with relevant national environmental standards and technical specifications, and compile a preliminary survey report, a detailed survey report, a risk assessment report, a risk control plan, a contaminated land remediation plan, as well as a governance and remediation evaluation report of contaminated land. The reports are uploaded and administered through the contaminated site information system and their main contents are open to the public. According to the recently released China’s Soil Pollution Prevention and Control Law’ in 2018, the state also implemented a system of risk management and remediation of soil pollution on construction land, where different stakeholders have different responsibilities [30].

### 3.3 Responsibility system for brownfield governance

A large number of existing brownfields in China have been formed after the relocation of old state-owned enterprises such as petrochemical and metal processing plants in the past. Some state-owned enterprises have been shut down or restructured, and the relevant responsibilities cannot be fully traced back. Even the existing ones cannot afford high repair compensation costs. Therefore, responsibility identification is one of the biggest problems in China’s brownfield governance. In the process of constructing an urban soil environmental management system, developed countries have continuously strengthened the unified supervision of central environmental authorities. At the same time, they have emphasised the appropriate decentralisation of central environmental management institutions, such as through the rational expansion of functions of local (provincial or regional) governments and the environmental administrative authority of the environmental protection agencies or through the establishment of branches directly under the central government, and full mobilisation of the local expertise in the governance of the local urban brownfields (i.e., municipal or county level government). Countries have stipulated in their legislation the responsibilities and authorities of relevant departments in detail, avoiding conflicts of power and interference with law enforcement. In terms of the competent authorities, the United Kingdom and the United States have granted strong enforcement powers to the environmental management departments to ensure that the polluters fulfil their obligations. They have paid attention to the division of responsibilities between the central and local governments and given full play to the initiative of local governments. The United States has given the USEPA powerful law enforcement powers, imposing heavy penalties on polluters and greatly improving the environmental protection awareness of enterprises. Similarly, the United Kingdom has given local environmental protection and health departments more comprehensive powers, including planning, investigation and administrative enforcement, and is planning to include all sectors related to the environment into the USEPA, ensuring a high degree of unity. Thus, the strength of law enforcement and the efficiency of execution have been improved. However, the relevant environmental enforcement departments are scattered in different departments and need to achieve unified management.

A national expert advisory committee has been recently established, which provides technical and specialist advice to the various stakeholders involved in brownfield assessment and redevelopment. The system requires a national set of soil contamination risk control standards and an accepted risk assessment scheme to be followed for urban brownfield redevelopment. Some cities may choose to modify these values for local purposes, but the national soil contamination risk control standards will serve as a baseline across the country to protect human health and crop production. The National Expert Committee can advise and instruct on governance issues and site investigations of relevant management departments, provide program support for risk assessment, reconstruction and post-reconstruction management, and propose key research areas and tasks. The expert committee should be composed of experts from various research fields and stakeholder groups. At the same time, an ‘Environmental Pollution Reconstruction’ or Brownfield Management Supervision Committee should work with the local environmental protection department to supervise and evaluate the risk assessment and remediation work at specific sites. The committee can exercise the rights conferred by the state, directly manage each member and supervise the relevant subordinate units, and form an effective program cycle chain (program establishment-program evaluation-implementation supervision-effect feedback).

On December 18, 2019, the MEE set up an Expert Advisory Committee on Soil Ecology and Environmental Protection, covering more than 60 people with different specialities in soil, groundwater, agriculture, and rural affairs. This group of experts serves as a think tank for advancing ecological and environmental protection in the fields of soil, agriculture, and rural areas and groundwater.

### 3.4 Brownfield governance fund and responsibility system

At present, China has no systematic legal provisions for the collection of taxes and fees for environmental losses/damage caused by soil pollution and has been establishing special funds, bonds, trust markets or other financing methods for brownfield redevelopment. Therefore, China also needs a financial support system for brownfield governance.

Although the issue of division of responsibility in the case of site contamination is not an easy task, most international regulations and policy frameworks adhere to the ‘polluter pays principle’. Experience in managing the US Superfund process has shown that:

• It is necessary to seek methods for determining the responsible party for pollution of sites with multiple discharges, such as landfills, and responsible persons for dumping sites.
• Effective methods must be sought to reduce the legal and administrative costs incurred by governments and small businesses.
• Management and law enforcement agencies need to consider the limited effectiveness of tracking those responsible for the inability to cover remediation costs.
• Site remediation is extremely expensive and must ensure a sustainable funding mechanism.

However, it is not the case in the United Kingdom, with the private sector promoting and funding most land development and rehabilitation projects. In some countries, the responsibility for contaminated sites is determined on a clear scale. The level begins with the polluter. If the polluter fails to pay the remediation cost, the responsibility will be transferred to the landowner. The transfer of responsibility to the government only applies if the landowner does not pay the remediation fee. In addition, there are special mechanisms for dealing with uninformed landowners’ responsibilities.

Under China’s policy framework, the basic principles of ‘polluter pays’ have also been clarified [31] as follows:

• The unit or individual that causes soil pollution shall bear the main responsibility for the control and restoration.
• If the responsible subject changes, the unit or individual who inherits its creditor’s rights or debts after the change shall bear relevant responsibilities.
• If the responsible subject is lost or the responsible subject is not clear, the people’s government at the county level shall bear relevant responsibilities according to law.
• Where the land use right is transferred in accordance with the law, the land use right transferee or the responsible person agreed upon by both parties shall bear the relevant responsibilities.
• If the land use right is terminated, the original land use right holder shall bear relevant responsibilities for the soil pollution caused during land use.
• The lifelong responsibility system shall be implemented to treat and mitigate soil pollution.

However, it is still necessary to further clarify the responsibilities of the various departments and comprehensively regulate the brownfield governance process. Measures for the Administration of Special Funds for the Prevention and Control of Soil Pollution [32] include (1) detailed investigation, monitoring and evaluation of soil pollution, (2) investigation and risk assessment of construction land and agricultural land, (3) prevention and control of soil pollution sources, (4) management and control of soil pollution risks, (5) remediation and treatment of soil pollution, (6) support to the establishment of provincial soil pollution prevention funds, and (7) enhancement of soil environmental supervision capabilities and other matters closely related to the improvement of soil environmental quality. The Ministry of Finance reviews and determines the amount of funding arrangements of the relevant provinces, autonomous regions, and municipalities in accordance with the allocation proposals made by the MEE [32]. According to the ‘Management Measures of Soil Pollution Prevention Fund’ released by the MEE in 2020, China has set up a soil pollution prevention fund at the provincial level. The fund has separate budgets or co-funding with social capital and adopts marketisation methods (such as equity investment) to exert guidance and leverage effects to guide capital investment for the prevention and control of soil pollution and government investment funds to support the development of the soil remediation industry.

### 3.5 Management of contaminated site remediation and the context of strategic city planning

In the practice of brownfield remediation, some common questions often arise: what level of soil pollution needs to be remediated? What is the target value for soil remediation? How to determine the target value of soil remediation? These questions are not only related to the quality of brownfield restoration but also directly related to the scope and the share of responsibility of the relevant stakeholders. At the same time, under the background of China’s ongoing industrial restructuring and strict control of urban and rural construction land, brownfield reuse has received widespread attention, but the current understanding of brownfield reuse remains unclear. Therefore, soil remediation goals and strategic planning for brownfield reuse are also important issues to be addressed in China today [33].

Early national policies emphasised multi-functional restoration (permanent contaminant removal). However, in most developed countries today, the overall trend of remediation tends to use ‘applicability’ as a target for remediation (i.e., the reused land needs to be ‘fit for purpose’ rather than return to a pristine condition). In other words, the required level of remediation/soil quality targets depends on the intended land use. The targets are generally classified into agricultural, residential and industrial/commercial uses. Site risk assessment and remediation objectives, therefore, usually need to consider the current or future land use [34].

For China, most brownfield industrial sites attracting attention are in cities, and many are in major real estate development areas. After redevelopment, these sites can be used for residential or commercial purposes to gain the greatest land price. Therefore, returning contaminated sites to their original uncontaminated state appears to be a conservative yet attractive option. However, many sites can have a pollution history of half a century or more, and given the time constraints of redevelopment, the time available for remediation is very limited. Expensive remediation costs and development time constraints can make it unrealistic to remediate contaminated sites to a standard applicable for all purposes. In addition, technologies that can effectively achieve rigorous remediation goals may not be available. Other potential land uses, such as industrial park sites or park green belts, may be more pragmatic and more economical options. Bardos et al. [35] (as cited in Ref. [36]) defined such reuses of brownfield sites as ‘soft reuses’, contrary to the ‘hard reuses’ based on built constructions or infrastructure, and suggested a ‘Brownfield Opportunity Matrix’ to understand the sustainability of the services and provide a structure for the overall valuation of restoration work.

The Chinese government has released a series of regional regulations regarding soil remediation [28]. The MEE has officially issued the technical guidelines for risk assessment of soil contamination of land for construction (HJ 25.3–2019). In 2018, China released its latest soil standards: soil screening values and intervention values. However, there is no clear remediation value released in China so far. The suitability, costs and time for various remediation technologies also require a system for independent testing, advice and verification. In recent years, a number of physical, chemical and biological treatment methods have been applied at brownfield sites, along with many claims for patents and commercially valued technologies. The scientific evaluation is critical for credible and feasible decisions over remediation targets and costs. Without this, the whole environmental engineering and remediation sector may ultimately be undermined.

## 4 Suggestions for future Chinese urban brownfield management and development

**Fig. 5:**
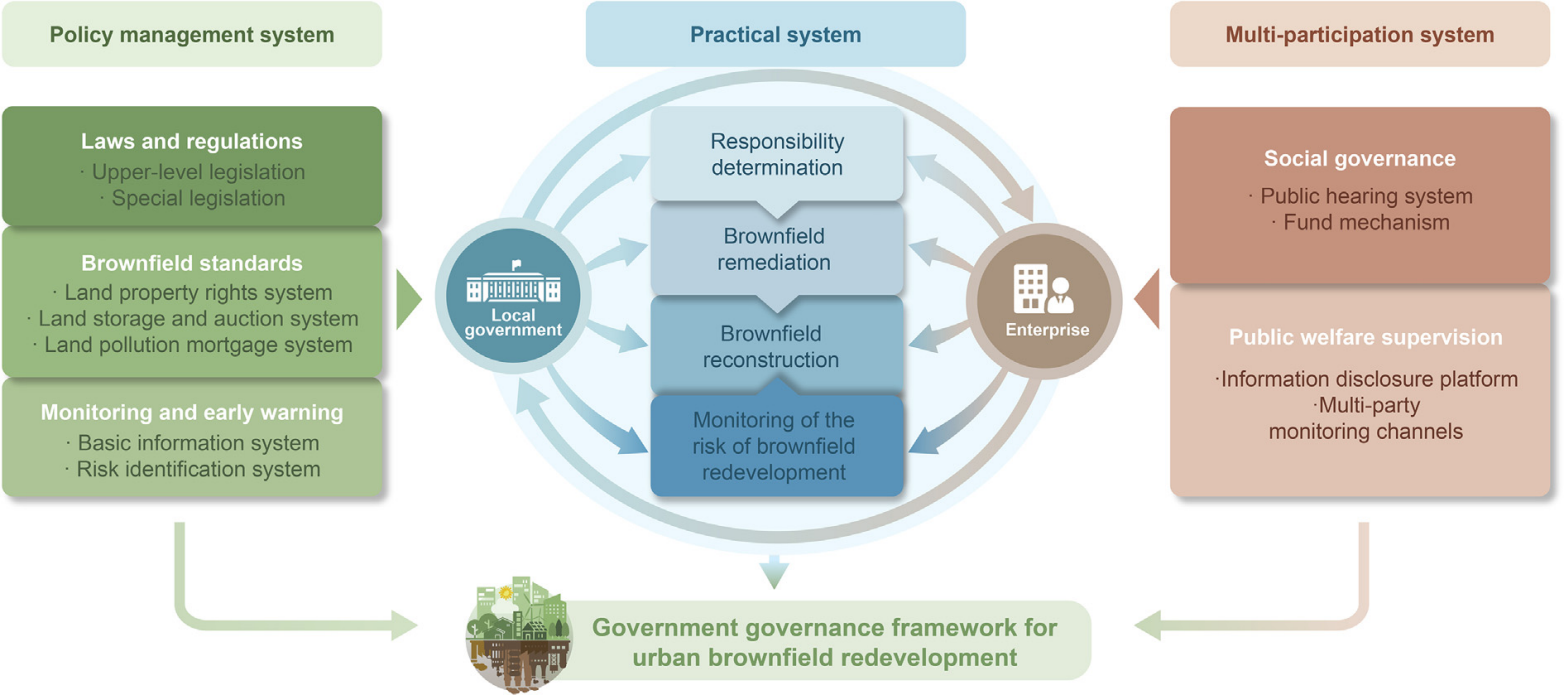
A proposed Chinese urban brownfield management framework (expanded from Ref. [38]). Green, blue and pink colours represent three systems and their relevance, respectively, which will serve the final goal—the government governance for urban brownfield redevelopment.

Although China is actively tackling soil pollution issues, there is still considerable room for strengthening the implementation of environmental policies and redeveloping brownfield sites [37]. Developing a coherent and integrated framework for brownfield management and redevelopment (Fig. 5) is an urgent and long-term strategic task for China. The timing is now optimal, as China is in an unprecedented stage of urbanisation and industrialisation. China has already invested and/or committed significant resources to implement brownfield redevelopment to promote eco-industrial and eco-friendly development.

While the United Kingdom and the United States have legislated the redevelopment of brownfield land as a systematic regional or national project, the specific situation of each brownfield site is different (e.g., the soil type, the contaminant mixture and level, the planned use of the site, etc.). Therefore, the general model is that the central government should guide the management and redevelopment of brownfield sites from a macro perspective. At the same time, the specific practical work should be promoted and managed by local governments and relevant stakeholders, according to local conditions and priorities (Figs. S3–S4). These experiences can serve as references for China, which has a stronger platform for control, informed decision-making and management than western models. China’s strong central and provincial planning capabilities and the single land public ownership system provide a huge opportunity for the overall planning of land use and site rehabilitation in the future.

First, China could use its powerful central legal system to formulate some standards for the governance and restoration of urban brownfields from both the upper-level legislation and the special legislation. On the basis of the system, the establishment of a registration system for the property rights of natural resources would provide a huge opportunity for the overall planning of land use and land restoration in the future. Besides, a soil pollution mortgage system also requires implementation. The amount of the mortgage for the treatment of contaminated land is estimated by a third-party professional organisation after the project is approved, and the payment of the mortgage is a necessary condition for the approval of the project. It can also initiate plans to use the land for specific purposes and benefits, such as providing renewable energy or developing soil-less three-dimensional agriculture. The government is responsible for authorising land use rights and providing guidance. In terms of capital investment, the central government’s fiscal investment is the main channel with an unfortunately huge funding gap. For this reason, the principles of ‘polluter pays’ and ‘beneficiary pays’ should be strictly implemented. The beneficiaries of the brownfield redevelopment process need to incorporate brownfield restoration into the planning and cost plan. It is also suggested to implement a strong monitoring and risk early warning mechanism, encourage sound technology, monitoring and on-site management, and control/authorise experts and professional institutions to use integrated monitoring methods of satellite, airborne and ground-based data, combined with space information systems to establish the basic brownfield information database, and set risk warning thresholds and response plans.

In terms of specific practices, local governments and related companies should maintain close communication. The first step is to determine the responsibility of the relevant subject that may be decentralised by the local government. Then, enterprises in the remediation and restoration of brownfields need to be helped and supported. On this basis, the brownfields can be redeveloped. Finally, the normalised management of monitoring and early risk warning will be jointly completed by the local government and enterprises.

Led by the Chinese government, a governance framework with multiple participation must be constructed. This includes social governance and public participation. A sound hearing system and multiple decision-making participation can help avoid the blind decision of managers and rent-seeking behaviour between governors and enterprises to a certain extent. Social funds and charitable organisations have supplemented the source of funds and raised public environmental awareness. Public supervision includes the construction of a transparent and effective information disclosure platform, the construction of multiple supervision channels and platforms such as the Internet, TV, newspapers and municipal Apps, allowing the public to raise questions, unblocking the communication channels between the public and the government, advocating public supervision and participation, and ensuring the smooth redevelopment of urban brownfields.

## Author contribution

YS wrote the first draft; KJ designed the study. All the authors contributed to generating and reviewing the subsequent versions of the manuscript.

## Declaration of competing interests

The authors declare that they have no conflict of interest.
