# Supplementary material for: Redevelopment of urban brownfield sites in China: Motivation, history, policies and improved management
Source: Eco Environ Health. 2022 May 21;1(2):63–72. doi: 10.1016/j.eehl.2022.04.005 (PMC10702914; doi:10.1016/j.eehl.2022.04.005)
Supplement: Multimedia component 1 [file mmc1.docx]

**Table S1:** The 10-point plan released in China in 2016.

| Plan | Content | Remarks relating to urban Brownfield |
| --- | --- | --- |
| 1 | Conduct soil pollution survey and master soil environmental quality | Deeply developing soil environmental quality survey; construction of monitoring network for soil environmental quality; Improving the information management level of soil environment. |
| 2 | Promoting legislation on prevention and control of soil pollution and establishing and perfecting the system of laws and regulations and standards | Speed up the legislative process; Systematic construction of standard system; Enhancing supervision and law enforcement in an all-round way. |
| 3 | Implementing classified management of agricultural land to guarantee the environmental safety of agricultural production | Classification of soil environmental quality of agricultural land; Enhancing protection effectively; Efforts to promote safe utilization; Fully implementing strict control; Strengthening soil environmental management of forest and grassland gardens. |
| 4 | Implementing access management of construction land to prevent habitat environmental risks | Clear management requirements; Implementing regulatory responsibility; Strict access to land. |
| 5 | Strengthen the protection of non-polluted soil and strictly control new soil pollution | Strengthening environmental management of unused land; Preventing new pollution of construction land; Strengthen the control of spatial layout. |
| 6 | Strengthen the supervision of pollution sources and preventing soil pollution | Strict control of industrial and mining pollution; Controlling agricultural pollution; Reducing domestic pollution. |
| 7 | Conduct pollution control and remediation to improve regional soil environmental quality | Define the subject of governance and restoration; Establishment of governance and rehabilitation planning; To carry out management and restoration in an orderly manner; Supervise the implementation of objectives and tasks. |
| 8 | Strengthen research and development of science and technology to promote the development of environmental protection industry | Strengthening the study of soil pollution prevention and control; Strengthen the popularization of applied technology; Promoting the development of governance and restoration industries. |
| 9 | Bringing the government's leading role into full play and constructing a soil environmental management system | Strengthening government leadership; Play a market role; strengthen social supervision; carry out propaganda and education. |
| 10 | Strengthen objective assessment and strictly investigate responsibility | Define the main responsibility of local government; strengthening department coordination and linkage; implementing corporate responsibility; Strict assessment and assessment. |

**Table S2:** Management documents related to contaminated sites issued by the state.

| **Date** | **Release agency** | **Laws/Regulations** |
| --- | --- | --- |
| 2001.12 | State Council | Reply on the National Environmental Protection Tenth Five-Year Plan |
| 2004.6 | State Environmental Protection Administration | Notice on Effectively Preventing and Controlling Environmental Pollution in the Process of Enterprise Relocation |
| 2004.8 | The eleventh meeting of the Standing Committee of the Tenth National People's Congress | Land Management Law of the People's Republic of China |
| 2006.2 | State Environmental Protection Administration in conjunction with the Ministry of Finance and the Ministry of Land and Resources | Guiding Opinions on the Progressive Establishment of Responsibility Mechanisms for Mine Environmental Governance and Ecological Restoration |
| 2006.11 | State Environmental Protection Administration | Notice on Issuing the “Eleventh Five-Year Plan” National Science and Technology Support Plan |
| 2008.6 | Ministry of Environmental Protection | Opinions on Strengthening Soil Pollution Control |
| 2009.8 | Office of the State Council | Guidance on strengthening prevention and control of heavy metal pollution |
| 2011 | Ministry of Land and Resources | Rules for the Preparation of Land Reclamation Plans |
| 2011.2 | Office of the State Council | "Twelfth Five-Year Plan" for Comprehensive Prevention and Control of Heavy Metal Pollution |
| 2011.03 | State Council | Soil reclamation regulations |
| 2011.10 | State Council | Opinions of the State Council on Strengthening Key Tasks of Environmental Protection |
| 2012.11 | Ministry of Environmental Protection and other four ministries | Notice on Ensuring the Environmental Safety of Industrial Enterprise Sites in Development and Utilization |
| 2013.01 | State Council | Notice on Printing and Distributing the Work Arrangements for Soil Environmental Protection and Comprehensive Management in the Near Future |
| 2014 | Ministry of Land and Resources | Regulations on Acceptance of Land Improvement Projects |
| 2014.3 | Office of the State Council | Guiding Opinions on Promoting the Relocation and Transformation of Old Industrial Zones in Urban Areas |
| 2014.4 | Ministry of Environmental Protection, Ministry of Land and Resources | National Survey Bulletin on Soil Pollution |
| 2014.4 | the eighth meeting of the Standing Committee of the Twelfth National People's Congress | Environmental Protection Law of the People's Republic of China |
| 2014.5 | Land Remediation Center of the Ministry of Land and Resources | Blue Book on Land Remediation |
| 2014.5 | Ministry of Environmental Protection | Notice on Strengthening the Prevention and Control of Pollution in the Process of Shutdown, Relocation and Redevelopment of Industrial Sites |
| 2014.12 | Ministry of Agriculture | National Announcement of Quality of Cultivated Land |
| 2014.12 | Ministry of Land and Resources | Announcement on the Results of Major Data Surveys and Assessments of National Cultivated Land Quality |
| 2016.5 | State Council | Action Plan for Soil Pollution Prevention |
| 2016.12 | Ministry of Environmental Protection | Measures for the Management of the Soil Environment in Contaminated Land |
| 2017.9 | Ministry of Environmental Protection, Ministry of Agriculture | Administrative Measures for Agricultural Land Soil Environment (Trial) |
| 2018.5 | Ministry of Environmental Protection | Industrial and Mining Soil Management Methods |
| 2018.8 | Standing Committee of the National People's Congress | Soil Pollution Prevention and Control Law |
| 2019 | Ministry of Ecology and Environment of People's Republic of China | National Environmental Benchmark Expert Committee established |
| 2019 | Ministry of Finance of the People's Republic of China and Ministry of Ecology and Environment of People's Republic of China | Measures for the Administration of Special Funds for the Prevention and Control of Soil Pollution |
| 2020. | Ministry of Finance, Ministry of Ecology and Environment, Ministry of Agriculture and Rural Affairs, Ministry of Natural Resources, Ministry of Housing and Urban-Rural Development, State Forestry and Grassland Bureau | Management methods for soil pollution control funds |
| **Date** | **Release agency** | **Standards of Soil Quality** |
| 1995.7 | Ministry of Environmental Protection | Soil environmental quality standard (GB15618-1995) |
| 2006.11 | Ministry of Environmental Protection | Environmental Quality Evaluation Standards for the Origin of Edible Agricultural Products (HJ332-2006) |
| 2007.6 | Ministry of Environmental Protection | Environmental Quality Evaluation Standards for Greenhouse Vegetable Producing Areas (HJ333-2006) |
| 2007.6 | State Environmental Protection Administration, General Administration of Quality Inspection and Quarantine | Evaluation Standards of Soil Environmental Quality for Exhibition Land (Interim) (HJ 350—2007) |
| 2013 | Ministry of Land and Resources | Land reclamation quality control standards (TD/T1036-2013) |
| 2015.8 | Ministry of Environmental Protection | Technical specifications for soil environmental quality assessment (draft for comments) |
| 2018.6 | Ministry of Environmental Protection | Soil Environmental Quality Agricultural Land Soil Pollution Risk Control Standard (GB15618-2018） |
| 2018.6 | Ministry of Environmental Protection | Soil Environmental Quality, Soil Pollution Risk Control Standards for Construction Land (GB36600-2018) |
| 2019 | Ministry of Ecology and Environment | Technical guidelines for risk assessment of soil contamination of land for construction (HJ 25.3-2019) |
| **Date** | **Release agency** | **Soil Remediation Technology Management** |
| 2014.2 | Ministry of Environmental Protection | Technical Guidelines for Site Environmental Investigation (HJ25.1-2014) |
| 2014.2 | Ministry of Environmental Protection | Technical Guidelines for Site Environmental Monitoring (HJ25.2-2014) |
| 2014.2 | Ministry of Environmental Protection | Technical Guidelines for Risk Assessment of Contaminated Sites (HJ25.3-2014) |
| 2014.2 | Ministry of Environmental Protection | Guidelines for Soil Remediation Technology of Contaminated Sites (HJ25.4-2014) |
| 2014.2 | Ministry of Environmental Protection | Terms of Contaminated Sites (HJ682-2014) |
| 2014.10 | Ministry of Environmental Protection | Contaminated Site Remediation Technology Catalog (First Batch) |
| 2014.10 | Ministry of Environmental Protection | Guidelines for Project Management of Contaminated Soil Remediation in Agricultural Land (Trial) |
| 2014.10 | Ministry of Environmental Protection | Guide to Extraction of Plants from Contaminated Soil of Agricultural Land (Trial) |
| 2014.11 | Ministry of Environmental Protection | Guidelines for Environmental Investigation, Assessment and Restoration of Industrial Enterprise Sites (Trial) |
| 2015.08 | Ministry of Environmental Protection | Guidance on Screening Guidance of Soil Pollution Risk for Construction Land |
| 2018.12 | Ministry of Environmental Protection | Technical Guidelines for Risk Control of Contaminated Land and Evaluation of Soil Remediation Effect |


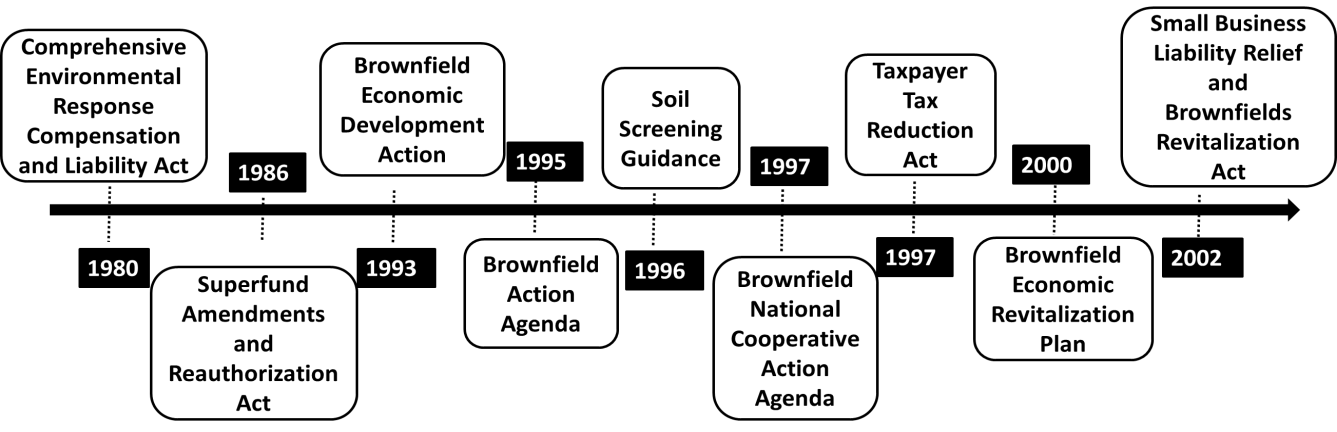


**Fig. S1:** Timetable of brownfield regulations in US


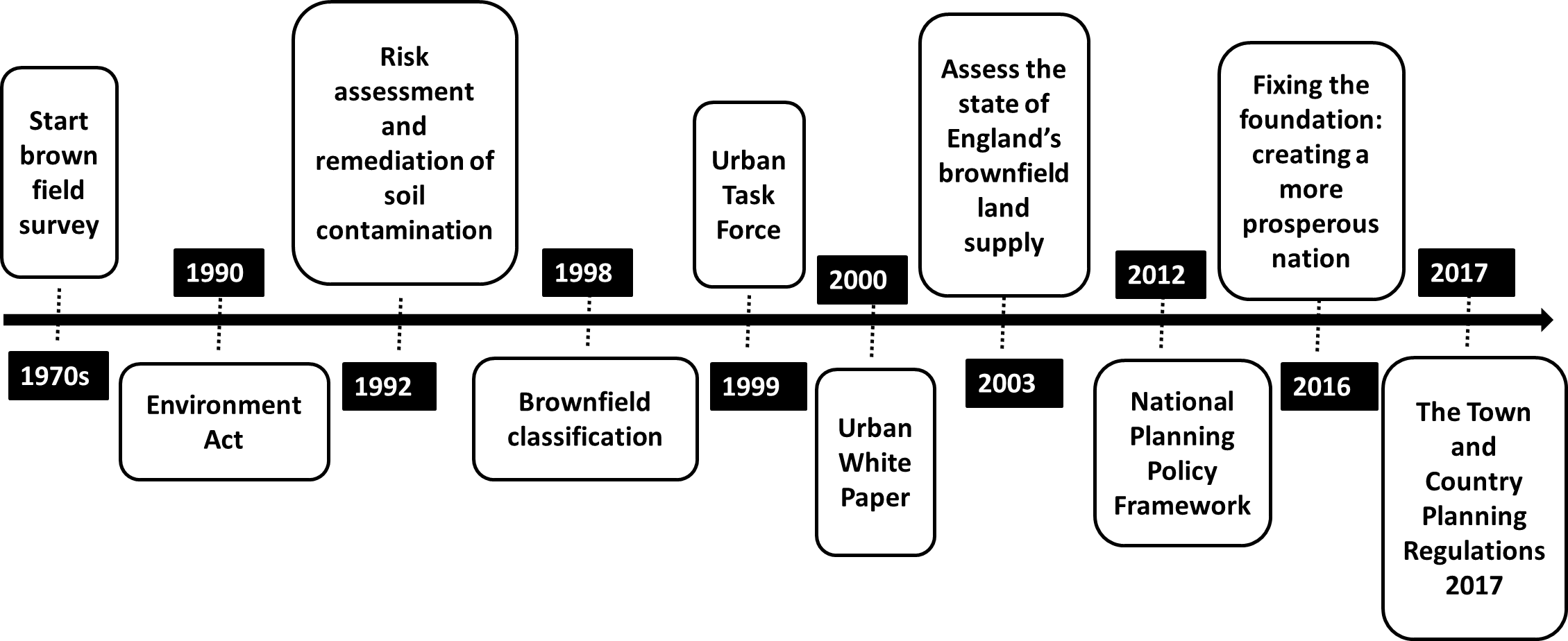


**Fig. S2:** Timetable of brownfield regulations in the UK


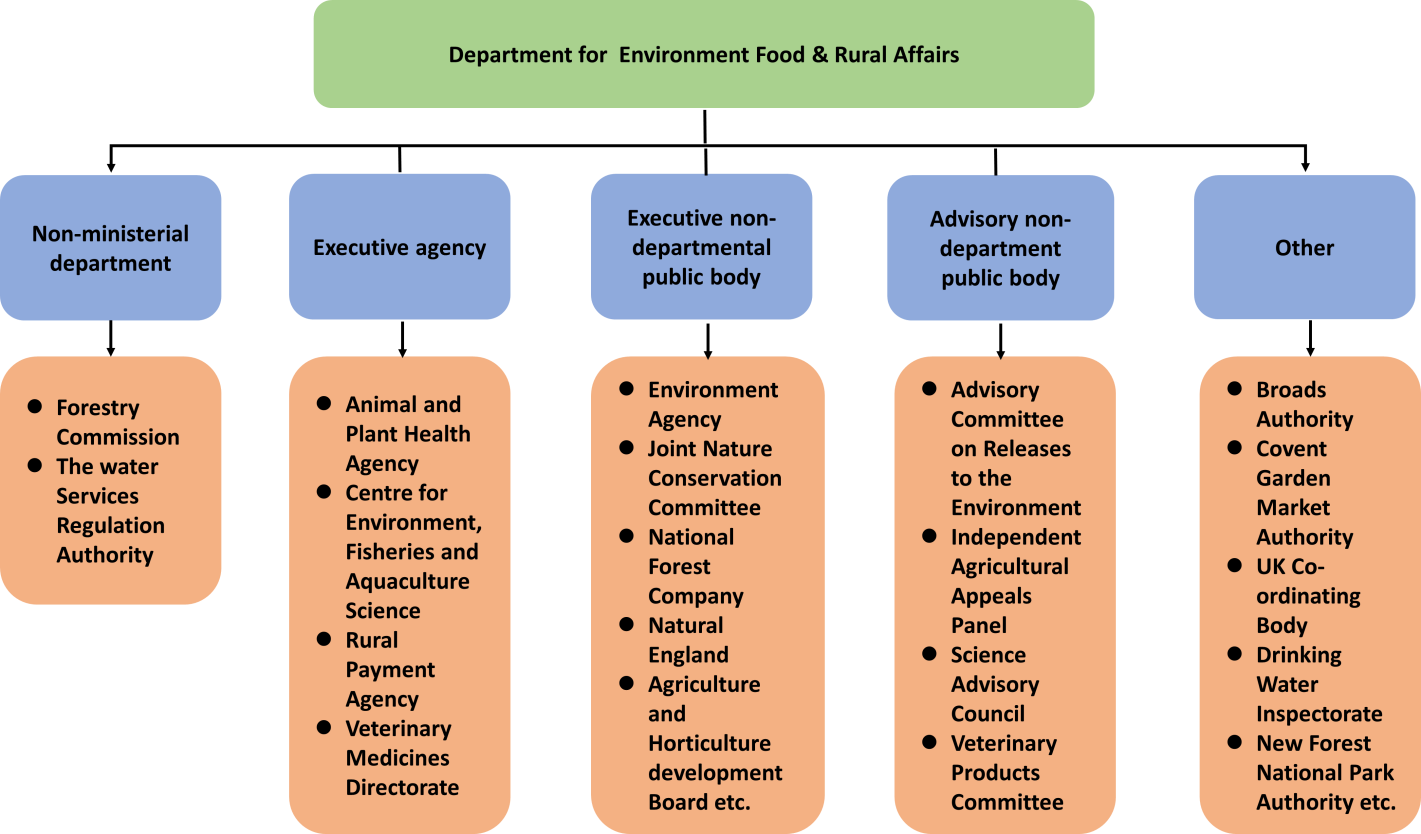


**Fig. S3:** The UK soil environmental governance framework


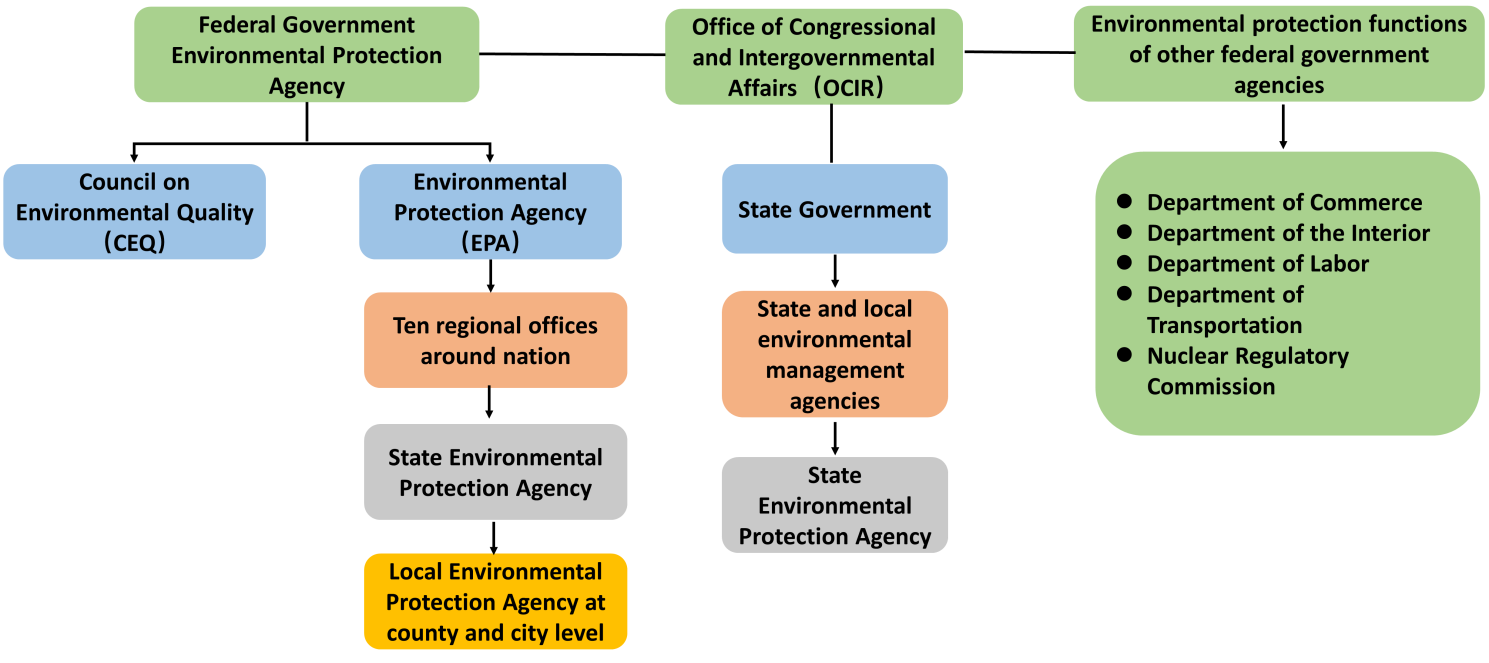


**Fig. S4:** The US soil environmental governance framework
